# Mobilization against forced domestic work in Peru

**DOI:** 10.3389/fsoc.2025.1504211

**Published:** 2025-03-28

**Authors:** Nicola Schalkowski

**Affiliations:** Institute for Latin American Studies, Freie Universität Berlin, Berlin, Germany

**Keywords:** domestic work, forced labor, ILO, Peru, trade union, vernacularization

## Abstract

Organized domestic workers have recently been at the forefront of political campaigns in Peru to promote legislative reforms concerning forced labor. This empirical case study investigates how the international labor norm concerning forced labor is disseminated, appropriated and politically mobilized at the national level. It thereby examines the dynamics and cultural embedding of the appropriation process and its implications for labor regulation, policy responses and trade union actions. The research delves into the ways and spaces in which international and national actors, the International Labour Organisation and national domestic workers’ trade unions, translate the international norms into their local context, adapt and negotiate them. I argue that the mobilization against forced domestic labor is embedded in the period of reconfiguration of the domestic work sector in Peru and that it is oriented toward internationally institutionalized categorizations and indicators. By employing the theoretical lens of vernacularization, a contextualizing examination of the labor policy actors, their positions and knowledge bases, as well as an analysis of decisive moments, spaces, technologies and developments of communicative actions, enables a sociological understanding of the emergence and manifestation of the mobilization against forced domestic work. The process of vernacularization creates a localized version of international norms that retains elements of the original while incorporating local specificities in the implementation. It creates a space where different forms of knowledge interact – local knowledge and transnational technicized knowledge. This research adds a nuanced understanding of the intersection between internationalization of ‘universal’ labor standards, knowledge production, and trade union mobilization within a feminized and racialized sector.

## 1 Introduction

Paid and unpaid domestic work is recognized as a deviation from the norm of standard employment on many dimensions due to its social arrangement within private households, outside of legally binding contracts and social security (see Schalkowski and Braig, 2023, p. 123, 130). This segment of labor markets is highly feminized and racialized and since it is constructed as a woman’s natural activity for social reproduction, it is often not considered work at all (Gutiérrez-Rodríguez, 2014; Anderson, 2000; Rollins, 1985). Domestic work does not fit into the idea of standard employment which is “an institution built around the permanently full-time employed male breadwinner working for a fixed employer” (Dingeldey and Gerlitz, 2022, p. 247) in industrial production. Likewise, labor relations that are labeled as ‘unfree’ or ‘forced labor’^1^ are considered to be a serious exception in the global economy and therefore a deviation from the standard employment norm (cf. Phillips and Mieres, 2015, p. 246) – even though coercive and violent labor relations are a structural and constitutive feature of the global labor market (cf. Van der Linden and Rodríguez García, 2016; Brass, 1999; De Vito et al., 2020).

When these two deviations from the norm intersect in the case of so-called *forced domestic work*, I speak of a *double deviation*. This perspective integrates the two categorizations and their interpretation patterns, opening up a new approach for sociological research.

Some social arrangements of domestic work, either in the Global South or involving migrants from the Global South, are observed as forced labor, domestic servitude or modern slavery (see e.g., Parreñas, 2015, 2021; Patterson and Zhuo, 2018; Mantouvalou, 2015; García Sedano, 2019). Domestic workers themselves have recently been at the forefront of political campaigns in Peru to promote legislative reforms concerning forced labor. In the streets of Lima in April 2021, members of the Federación Nacional de Trabajadores y Trabajadoras del Hogar Remunerados del Perú (FENTRAHOGARP) posed for a newspaper holding signs saying “NoMásTrabajoForzoso” (NoMoreForcedLabor), “Un futuro libre de trabajo forzoso” (A future free of forced labor) and “Trabajo decente para todas y todos” (Decent Work for everyone), (El Peruano, 2021). Even given a informality rate of 82.3, 14.6% of domestic workers working more than 60 h a week (OIT, 2021, p. 39), insufficient rest and payment, degrading accommodation and a high rate of arbitrary abuse and violence (Defensoría del Pueblo, 2016; Maich, 2014, p. 86; OIT, 2013), labeling cases of severe labor rights violations and servitude-like living and working conditions of domestic workers as ‘forced labor’ is new. Until recently (2003), paid domestic work was not even regulated since it was not considered employment in Peruvian labor legislation.^2^ Interestingly, for this campaign the trade unionists prominently displayed a publication by the International Labour Organisation (ILO) on forced labor in the domestic work sector in Peru (OIT, 2018). The activists thus addressed the Peruvian government and made a direct reference to a legal framework and classification system which was defined at the international level of the ILO and transferred it to their local context.

Forced labor was defined as a relevant area of concern requiring legal regulation and political intervention in 2007 in Peru’s first National Plan for the Fight against Forced Labor (Ministerio de Trabajo y Promoción del Empleo, 2007a). A few years later, the Ministry of Labor named three focus areas to combat forced labor in the country: wood cutting, mining and domestic work (Ministerio de Trabajo y Promoción del Empleo, 2013a). In what context and on what basis did the focus on forced domestic work come about – after all, the sector is highly invisible and informally organized, has a low organization rate and mainly women of low social status performing the work? This empirical study investigates how the international labor norm concerning forced labor is disseminated, appropriated and politically mobilized at the national level. It thereby examines the dynamics and cultural embedding of the appropriation process and its implications for labor regulation, policy responses and trade union actions. The mobilization against forced domestic work at the legal, governmental and trade union level in Peru form the empirical case for this endeavor. Building on historical-sociological studies on international organizations (Kott, 2011; Caruso and Stagnaro, 2017; Plata-Stenger, 2020; Sinclair, 2017; Maul, 2007) and drawing on insights from critical sociology of knowledge (Berger and Luckmann, 1969), this investigation contributes to the debate on labor market segmentation by applying a reflexive perspective on categories of work and their political mobilization.^3^

In this endeavor, I investigate wider questions through the perspective of the sociology of knowledge, methodically concentrating on the observational patterns applied to the field of work, concerning violent and coercive forms of work as well as the cultural framing of household, family and freedom. Furthermore, from a historical-sociological viewpoint, I examine the international regulation of work and the mobilization of labor norms at national level.

I argue that the mobilization against forced domestic labor is embedded in the period of re-configuration of the domestic work sector in Peru and that it is oriented toward internationally institutionalized categorizations and indicators. The latter supplement national norms and relate to normative concepts of labor and unfreedom that create interpretive frameworks. The research delves into the ways and spaces in which international and national actors translate the international norms “into local terms” (Levitt and Merry, 2009, p. 445), adapt and negotiate them. Overall, “we segment the world of work” (Renard and Wobbe, 2023, p. 105) by using categories, which has consequences for labor market regulations and configurations as well as political fields of action.

Methodologically and analytically, my empirical study draws on Sally Engle Merry’s vernacularization approach (Merry, 2006a; Levitt and Merry, 2009), which enables a sociological examination of social processes “of appropriation and local adoption of globally generated ideas and strategies” (Levitt and Merry, 2009, p. 441). This perspective attempts to bridge the presumed contradiction between universalism and relativism concerning international human rights implementation on national and local levels (Merry and Levitt, 2017, p. 213). Analyzing the mobilization against forced domestic work as a process of vernacularizing an international labor rights standard makes it possible to relate local norms, actors and policy practices to transnational contexts in which categories and norms are set. This is not a process of simple top-down diffusion of the ILO category of forced labor on a legal dimension, but a process of translation, adoption and local negotiation of interpretation patterns of violent and coercive (labor) relations on different dimensions. The process of vernacularization creates a localized version of international norms that retains elements of the original while incorporating local specificities in the implementation. It creates a space where different forms of knowledge interact – local knowledge and transnational technicized knowledge.

The article is structured in six steps. In sections 2 and 3, I provide a discussion of the theoretical approach of vernacularization and an illustration of the empirical-methodological procedure. Fourthly, in the findings section, I start by discussing the contextual conditions that facilitated the debate on forced domestic labor. This is followed by an illustration of the ILO’s technical assistance and labor advocacy approach to localizing the category into Peruvian contexts, and the appropriation and mobilization on the trade union level. In the fifth step, the key findings are discussed concerning the dynamics and cultural embedding of the vernacularization process and its implications for labor regulation, policy responses and trade union actions. In the outlook I not only reflect on the implications of my analysis for future research but also identify political implications.

## 2 Theoretical approach to transnational norm building and local appropriation

With this case study, I analyze how the ILO’s legal category of forced labor, defined at the transnational space of the International Labor Conference in 1930, and its respective interpretation and classification patterns (indicators) “are vernacularized to fit particular historical and social contexts, thereby producing shared notions” (Levitt and Merry, 2009, p. 443) about coercive and violent relations and practices at work. The empirical as well as theoretical concept of vernacularization is based on Sally Engle Merry’s anthropological research in the early 2000s on the implementation and local practice of women’s rights within a framework of human rights (Merry, 2006a). This concept is productive in the study of how international norms are translated and implemented in different cultural and social settings and “become part of local social movements” (Merry, 2006a, p. 134). The interplay between global standards and local realities is mediated by various actors who bridge these different contexts. This process involves more than mere linguistic translation; it requires adjusting global ideas to align with local values, beliefs, and social structures. Merry and Levitt empirically observe, that in the adaptation process global norms are not merely imposed from above but are negotiated and reframed to resonate with local contexts, ensuring that universal principles can be meaningfully integrated (Levitt and Merry, 2009, p. 446).

How do the ILO’s standards around forced labor “connect with a locality” (Levitt and Merry, 2009, p. 446) in Peru? This happens through actors in various positions and communicative actions on various levels, e.g., within public policy plans, video campaigns, trade union actions, sector-specific training or in conversations with affected workers. Vernacularization is determined by the communicators’ position within the social hierarchy and their respective institution, technologies and channels of transmission, historical-cultural geographies in which vernacularization takes place, as well as the content and embeddedness of the ideas which generated at the global level (Levitt and Merry, 2009, p. 446). To fully realize an emancipatory or transformative potential for the domestic workers’ organizations, concepts and classifications of violence and coercion must become part of the “local legal consciousness” (Merry, 2006a, p. 179).

In this case study, two relevant interacting groups are observed: ILO specialists and trade unionists (respectively organized domestic workers). *These actors “convey ideas from one context to another, adapting and reframing them from the way they attach to a source context to one that resonates with the new location.”* (Levitt and Merry, 2009, p. 449). The source context, or better *contexts*, are the contexts of categorization at the transnational ILO level negotiated by representatives of national governments, workers’ organizations and employers’ organizations,^4^ while the new location is the national/local context of domestic work in Peru. A significant contribution of this study is to link the “global production and [the] local appropriation” (Merry, 2006a, p. 6) of a labor norm. It highlights the connection between the historic-geographical context-dependent patterns of norm setting which shape the understandings and the regulation of worlds of work, and the communicative actions of national actors in dialogue with international organizations’ actors. A central challenge in the application of international rights is the tension that arises between universally formulated standards – based on institutionalized technical knowledge – and local particularities. International organizations are “sites of internationalisation” (Kott, 2017, p. 34) where, in the consensus building discussions on categorizations and norms, knowledge circulates between various international actors. For the sake of universal comparability and applicability, the ascribed transnational experts focus on general principles disregarding specificities of local situations. Merry describes this process as a “cultural practice” (Merry, 2006a, p. 228) in which “actors come together simultaneously as locally embedded people and as participants in a transnational setting that has its own norms, values, and cultural practices.” (Merry, 2006a, p. 37). Through the continuous exchange of observations and proposals, the understanding and range of a phenomenon as well as the international framework of action (policy script) is determined by introducing their specialized technicized (and de-politicized) knowledge and classification patterns into political decision-making processes.

At the ILO, the world of work is objectified through a specific technical language, and the production and reproduction of knowledge about it leads to a structural consolidation of interpretative patterns (Berger and Luckmann, 1969): “the ILO is a definitional arena, promulgating what counts as work and who is a worker for the world.” (Boris, 2019, p. 2). These patterns find an institutionalized expression in the ILO’s legal and statistical framework, transmitted in technical assistance missions (see Plata-Stenger, 2020; Kott, 2017).^5^ The ILO’s category of forced labor is an illustrative example. What can be understood and legally condemned as forced labor was established by the International Labour Conference (ILC) in 1930 with the adoption of the Forced Labour Convention (No. 29). Convention Nr. 29, still in force today, defines forced labor as “all work or service which is exacted from any person under the menace of any penalty and for which the said person has not offered himself voluntarily” (Article 2(1)). The drafting of Convention No. 29 emerged from the deliberations of a Committee of Experts on Native Labour^6^ and discussions at the ILC. The regulation of forced labor was embedded in the cultural framework of the European civilizing mission within the imperial context of the interwar period (Wobbe et al., 2023). The controversial discussions drew on a specific ‘colonial knowledge’ base (Schalkowski and Renard, 2024, p. 593) and revolved around “extra-economic coercion” (Breznik, 2023, p. 1126). Forced labor was established as a colonial phenomenon and defined against the criterion of free male industrial wage labor, distinguished from slavery and specified in terms of colonial and gender differentiations (Wobbe et al., 2023, p. 174; see also Rodet, 2014). It entails gender-specific implications based on the feminization of certain activities that were defined as “communal services” and excluded from the reference field of (forced) labor. Furthermore, women at workplaces were invisibilized by addressing them as ‘companions’, ‘wives’ or ‘family’ of working men and not as workers themselves (Rodet, 2014; Wobbe et al., 2023). This gendered differentiation “*introduced the distinction between a so-called traditional, community-related private sphere and the so-called public sphere of labour*.” (Wobbe et al., 2023, p. 184).

I argue that specifically the constructed separation of the public and the private sphere has shaped the gendered patterns of interpretation and classification of work. Despite the ILO’s shift toward universalist human rights principles of labor over the course of the 20th century (Maul, 2007; Renard and Wobbe, 2023) and recent attention to coercive practices in domestic work, the focus remains on market-based work and the “male baseline of international labour regulation” (Vosko, 2009, p. 67) persists. Consequently, non-market-based reproductive activities in the household do not fall within the interpretative framework of the category of work, and thus consequently also not within that of forced labor (see Schalkowski and Renard, 2024).

Understanding these distinctions as relational and contingent in their expressions and classifications in different historic and institutional contexts implies looking at how interpretive patterns are (re)produced and adapted by different actors and institutions (e.g., on the national/local level). Vernacularization is a critical concept which highlights the complex and dynamic process through which international standards are adapted to fit local contexts, mediated by various actors and influenced by local social and cultural factors.

## 3 Material and method of analysis

The study employs a qualitative, reflexive approach, integrating document analysis and expert interviews with domestic workers’ unions and ILO specialists, complemented by interviews with relevant actors in the field such as feminist and labor rights activists, labor lawyers and social scientists. The anonymized interview material was conducted between 2020 and 2023 in the framework of a multi-year research project. The interviewees are considered experts because they have acquired specialized knowledge through their occupation, status or position and therefore have privileged access to information (Bohnsack et al., 2018, p. 76). This methodological approach makes it possible to understand the positions, roles and relationships of the international and local actors as well as their interpretations, appropriations and negotiations of the institutionalized norms on forced labor. It further allows us to identify focal moments, spaces, technologies and developments of communicative actions that have shaped the mobilization against forced labor.

The interviewed domestic workers’ trade unionists and activists are organized in: AMUNETRAP,^7^ FENTRAHOGARP,^8^ FENTTRAHOP,^9^ IPROFOTH,^10^ SINTTRAHOL^11^, and SINTRAHOGARP.^12^ ILO specialists included project coordinators from the Special Action Programme to Combat Forced Labour (SAP-FL) in Lima and Geneva, and Domestic Work ILO Programmes in Peru, a former trade union specialist (ACTRAV),^13^ and ILO communication staff. Other contacts with activists on the ground (from Casa de Panchita,^14^ Centro de la Mujer Peruana Flora Tristan^15^ and ANTRAH^16^) came about through conversations with trade unionists and the ILO staff, or at trade union and ILO events in Lima.

For the review of archives, the primary sources included relevant public documents and communication materials from the ILO and the trade unions. Moreover, I analyzed public documents from the Ministry of Labor, such as session protocols from the National Commission for the Fight against Forced Labor and the National Plans against Forced Labor, since they are the product of recurrent negotiations, which set the framework for legislation and policy interventions. In addition, I considered the Peruvian Law on Forced Labor, the UN Report on Contemporary Forms of Slavery in Peru (2011), as well as other relevant studies on forced labor and domestic work in Peru.

The analysis of the material is methodically guided by Braun and Clarke’s reflexive thematic analysis (see Braun et al., 2018). Initially, the data was processed inductively by openly coding the contextual sense-making of the interviewees in relation to their observations and interpretations of processes and experiences. In the following analysis of the data themes that serve as “a central organizing concept” (Braun et al., 2018, p. 3) were generated to highlight the connections within the data and patterns of meaning that relate to the research question. The two themes of ‘Internationalization of the forced labor category through labor rights advocacy’ and ‘Trade unions’ mobilization through appropriation of the category and resource mobilization’ are embedded in the theoretical framework of vernacularization. This approach, as outlined by Braun and Clarke (Braun et al., 2018), is a qualitative research approach that emphasizes the researcher’s reflexivity and the active engagement with the data.

## 4 Mobilization against forced domestic work

The mobilization against forced domestic work in Peru is embedded in specific historical-cultural contexts which enabled the establishment of the issue on the political agenda (4.1). Analyzing this development as a process of the vernacularization of the ILO norms reveals, on the one hand, the ILO’s labor rights advocacy approach to facilitate the internationalization of the forced labor category (4.2), including the production of knowledge (4.2.1) and the development of an institutional structure (4.2.2), which find their expression in information and educational campaigns (4.2.3). These processes involve translating the abstract principles of international labor standards into concrete terms that resonate with the Peruvian ministries and with the lived experiences of domestic workers. On the other hand, during the process of vernacularization the organized domestic workers appropriate the category of forced labor and are not only translators but also affected workers (4.3.). They reframe known situations into the ILO’s paradigm (4.3.1), whereby they self-identify as victims of forced labor (4.3.2), and use the newly established norms as a resource (4.3.3).

### 4.1 Forced labor as a political field of action in Peru

Coercive and violent labor relations were categorized as forced labor and thus became visible in Peru in the 20th century mainly through trade unions and official claims of the Committee of Experts on the Application of Conventions and Recommendations (CEACR) of the ILO. The Peruvian government ratified ILO Convention No. 29 in 1960, and since then, the ILO’s CEACR has periodically reviewed and commented on its application. Initial CEACR reports focused on state-exacted forced labor, such as compulsory military service (1960s) and compulsory prison labor (1970s-1990s), mentioning in particular the discrimination against indigenous peoples. Regarding the private sector, the committee’s observations likewise recognized shortcomings in legislation that allowed forced labor to be imposed on indigenous communities, especially regarding colonial continuities in agriculture (observed in the 1960s and 1980s). In his compliance study on the application of international labor standards on forced labor, Thomann (2011) suggests that in the democratic period which followed the restrictive Fujimori regime of the 1990s, the Peruvian government found “normative guidance” (Thomann, 2011, p. 268) in the juridical observations and comments of the CEACR, and that returning “to a compliant behavior [was] a signal towards other actors that Peru again was a reliable partner in international relations that stood up to its commitments.” (Thomann, 2011, p. 268).

The CEACR did not report forced labor in domestic work until the 2000s when it referred to the Workers’ General Confederation of Peru’s (CGTP) concerns about

“*Women workers who are exploited and obliged to work over 18 h a day without receiving remuneration, or with their remuneration being paid in kind, and who are deprived of their freedom of movement or their identity papers*.” (ILO, 2011b).

That the male dominated trade union confederations, who had ignored the domestic work sector (Blofield and Jokela, 2018, p. 537; Blofield, 2009, p. 184), recognized the severe working conditions as a labor policy issue was due to a significant transformation of the trade union landscape and international discourse. Peru saw increased organization among domestic workers in the late 2000s and early 2010s, notably with the formation and recognition of SINTRAHOGARP in 2006, SINTTRAHOL in 2009, and FENTTRAHOP in 2012. The mobilization for international labor regulation of domestic work sparked this wave of organization (see Boris and Fish, 2014; Blackett, 2019), since formal worker organization became essential for tripartite negotiations at the ILO in Geneva. The campaigns raised awareness globally and nationally, leading to the Domestic Work Convention No. 189 in 2011 (ILO, 2011a), which enabled a reconfiguration of labor markets since it formally recognized, defined, and regulated domestic work. It was in this context that a UN mission reported on domestic servitude, in the framework of contemporary forms of slavery in Peru, recommending more research and legal regulation (UN, 2011).

However, according to the domestic workers’ unions and ILO staff, it was in the context of the COVID-19 pandemic when the issue of forced domestic work gained momentum. The Peruvian government reacted with a series of rigorous lockdowns spanning several months, which put a spotlight on invisible work arrangements in households. The severe working and living conditions of domestic workers (especially those from rural areas) received increased public attention and were scandalized (see e.g., Pereyra Colchado, Gladys, 2020; IUS Latin, 2021; Rosas, 2020), and framed as forced labor:

*“In the time of the pandemic, they started talking about forced labor. […] there was a lot of talk because there have been many domestic workers locked up, just receiving food, because the employer told them that they do not have money to pay them. […] The pandemic has highlighted the degree of vulnerability in which we domestic workers find ourselves.”* (Delia M., Trade Unionist).

Surveys and studies, employing quantitative as well as qualitative methods, were initiated by the workers’ organizations who were highly concerned with the psychological and physical conditions of their colleagues. They distributed online questionnaires on domestic workers’ health and working conditions during the lockdown periods (FITH, 2020; SINTTRAHOL, 2021). These debates prompted the regulation of a new national law on domestic work (Law 31047, 2020), which the trade unions had been fighting for since the adoption of ILO Convention 189 in 2011 and which includes protection against forced labor (Law 31047, Article 22).

### 4.2 ILO labor rights advocacy – internationalization of the forced labor category

The ILO’s vernacularization efforts operate within the framework of an international labor rights advocacy (cf. Merry, 2006a, p. 164) to internationalize its standards. This includes conducting studies or surveys, setting up institutional infrastructure such as legislation and commissions, creating information material, awareness-raising campaigns and educational training programs. These technologies, which constitute an integral element of the ILO’s technical assistance for development cooperation, strategically facilitate the internationalization and legitimacy of their category, secure funding (in this case from the U.S. Department of Labor USDOL) and offer a heuristic benefit regarding the production of knowledge on local or general sectoral manifestations of coercive and violent labor practices. These activities are embedded in the international policy script of the ILO concerning forced labor and are possible due to the resource-rich presence of the ILO in Peru.^17^ Within the international bureaucratic structure of the United Nations, the ILO is an international instance of observation and regulation of the world of work and operates as a depoliticized actor.^18^ As a specialized agency of the UN, the ILO provides technical assistance to both governmental institutions and trade unions (historically for Latin America, see for example Plata-Stenger, 2020; Caruso and Stagnaro, 2017). Together with Brazil, Peru is currently the center of ILO activities for the elimination and prevention of forced labor in Latin America.

The renewed policy script concerning forced labor was established in the late 90s and early 2000s. In 1998, the ILO’s binding Declaration of Fundamental Principles and Rights at Work incorporated the elimination of forced or compulsory labor as one of four core principles and rights, linking labor rights to universal human rights (ILO, 1998). Following up, the ILO’s Governing Body instituted the Special Action Programme to Combat Forced Labour (SAP-FL) in 2001 as a first structural mechanism to address the issue (Plant and O'Reilly, 2003). Moreover, the ILO’s Global Report (ILO, 2001) identifies ‘modern’ types of forced labor, including domestic work (ILO, 2001, p. 2; see Maul, 2007, p. 494), highlighting it as one of eight “special issues for further action” (ILO, 2001, p. vi, 103). The ILO’s activities in Peru are part of this global program. Thomann observes the legislative developments in Peru and argues that the incorporation of forced labor in the nation’s political discourse would likely not have occurred without the initiatives of the ILO’s SAP-FL (Thomann, 2011, p. 280). Following the adoption of a new instrument to address the so-called ‘modern’ forms of forced labor – the Protocol of 2014 to the Forced Labour Convention No. 29 (ILO, 2014) – a technical assistance program to facilitate its ratification was created. In 2015, “From Protocol to Practice: A Bridge to Global Action on Forced Labor (The Bridge Project),” funded by the USDOL, started in Peru as part of the SAP-FL.^19^ In two phases (so far) the project has the mission to increase knowledge and awareness, implement the Forced Labour Protocol into national legislation (phase I), as well as to build capacities of relevant actors (such as lawyers, trade unions and labor inspectors) for the application of the Protocol (phase II). For a chronological overview of events concerning forced labor policies on an international and Peruvian level (see Table 1).

**Table 1:** Chronology, localizing the ILO’s forced labor category.

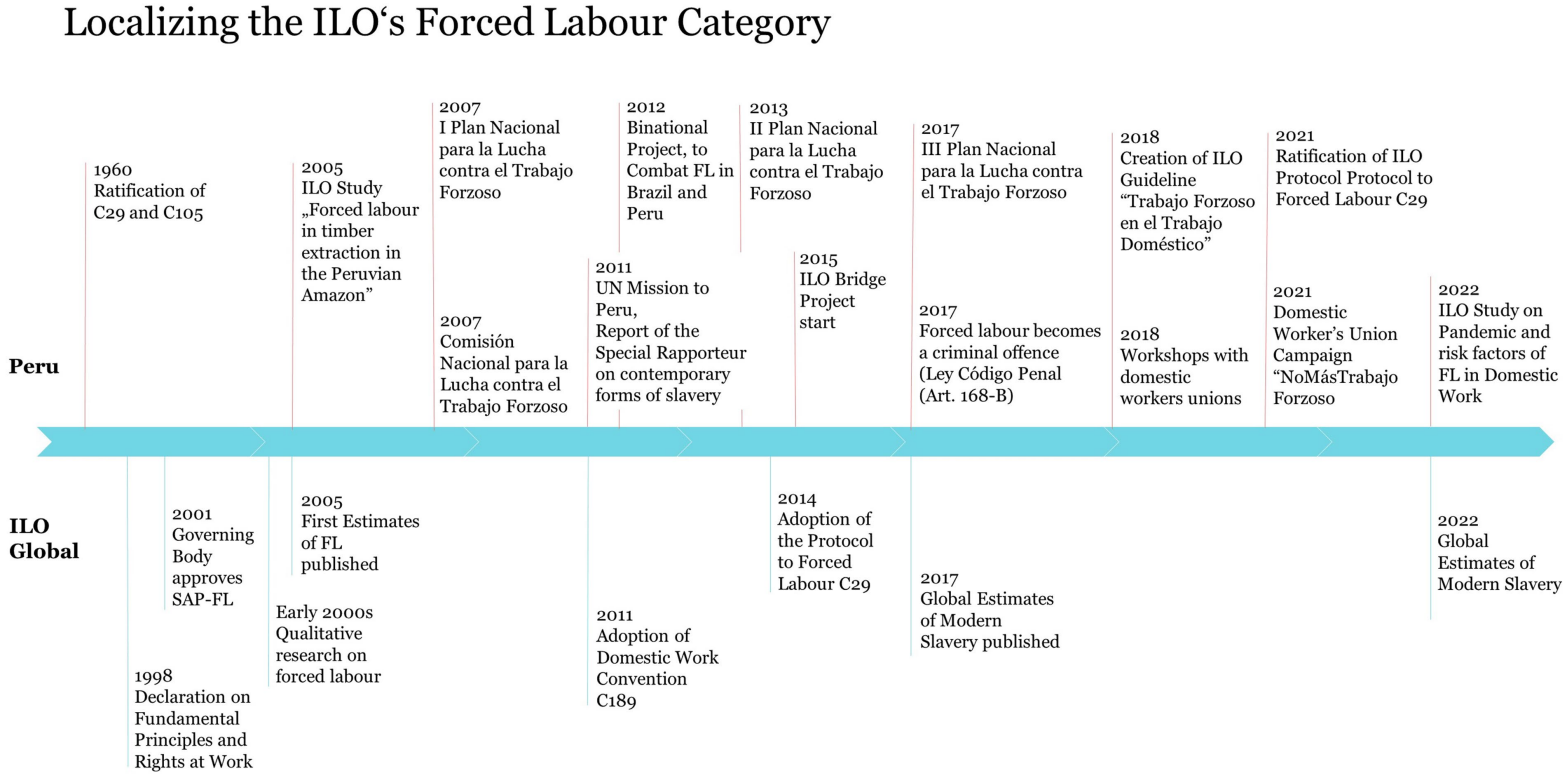

#### 4.2.1 Knowledge production

Prior to considering the formulation of an agreement on technical assistance to support the Peruvian government in combating forced labor, it was imperative for the ILO to establish an empirical knowledge base on the issue beyond the legal observations of the CEACR. Three studies were mentioned as particularly relevant by the interviewed actors as well as in government documents. In 2005, an impactful qualitative study by anthropologist Bedoya Garland and social scientist Bedoya Silva-Santisteban on forced labor in the timber industry in the Peruvian Amazon, commissioned by the ILO, served as crucial evidence of forced labor’s existence (Bedoya Garland and Bedoya Silva-Santisteban, 2005). The ILO workers’ specialist states that this investigation was not a mandate from the Peruvian government but the ILO headquarters in Geneva.^20^

*“It even cost, let us say, an effort for the ILO to make the publication, because the government did not recognize the existence of forced labor in Peru.”* (ILO Workers’ Specialist).

The empirical cases were predominantly related to indigenous populations and substantiated the CEACR observations on debt bondage and forced labor in the Amazon region. The discussion of the results was the decisive moment for the government’s commitment to a structured approach to addressing the issue (Ministerio de Trabajo y Promoción del Empleo, 2023, p. 6). The Amazon border region with Brazil and Bolivia was again at the center of knowledge production in an ILO study from 2015 (Sanz, 2015). Once more, the subject was a productive sector in which mainly male rural and/or indigenous workers are found: mining. Subsequently, based on the findings of these studies, timber harvesting and mining were prioritized for measures to eradicate forced labor in Peru. These investigations are based on the ILO’s legal category of forced labor and generally lack an appropriate gender analysis. Women’s activities are hardly mentioned and only in some cases are they linked to the forced labor of men. The mining study, for example, mentions women in ‘service’ surrounding the mines, but does not investigate the issue:

*“The study allows us to show one of the groups mainly affected by forced labor: the immigrant population in the highlands, as well as women in mining-related services.”* (Sanz, 2015, p. 7; own translation) [*…*] *“the analysis of what happens in these services was not part of the research.”* (Sanz, 2015, p. 38; own translation).

Here, women’s activities are excluded in the production of knowledge, preventing an analysis of the possible connections or entanglements between the coercive labor of men and that of women at a workplace or a value chain.^21^ A particular and historically narrow concept of labor (SER), reflecting the standard labor norm of a male industrial wage worker, becomes evident. Which social arrangements fall within the frame of reference of (forced) labor, and are thus subject to legal and political interventions, is influenced by the gender-biased production of knowledge.

To date, there is no comparable ILO study on forced domestic work in Peru.^22^ Nonetheless, the Peruvian authorities named the domestic work sector in the National Action Plan II as the third focal sector which requires immediate action (Ministerio de Trabajo y Promoción del Empleo, 2013a), even though it has “not been addressed by the CEACR or the Conference Committee which evidently lacked the necessary information to address the topic.” (Thomann, 2011, p. 278). As the only knowledge base, the Action Plan cites a 2011 report by the UN Special Rapporteur on Contemporary Forms of Slavery that presents conditions of domestic work in Peru as “domestic servitude” (UN, 2011, p. 3) concerning especially rural-born young women or children working in urban households. On an international scale, the ILO’s first statistical Global Estimates of Forced Labour (ILO, 2005b) named forced domestic work as one of the most dominant forms of private forced labor in Latin America and the Caribbean (ILO, 2005b, p. 3).

#### 4.2.2 Institution building

Another aspect of the vernacularization of the ILO norms on forced labor within labor rights advocacy, besides the “localization of transnational knowledge” (Merry, 2006a, p. 20), is the building of an institutional structure. In the case of Peru, three milestones were established as a result of communicative action within the framework of the ILO’s technical assistance: a national commission, the national action plans, and the law against forced labor. The National Commission for the Fight against Forced Labour (Comisión Nacional para la Lucha contra el Trabajo Forzoso CNLCTF), established in 2007, creates and monitors the institutionalized governmental strategy in the form of National Action Plans (Planes Nacionales para la Lucha contra el Trabajo Forzoso). Following the tripartite logic of the ILO, the commission consists of representatives from several ministries, employers’ associations and workers’ organizations. This newly created structure could therefore be used by the trade unions as a platform to showcase their concerns and priorities. However, in 2007, for example, no representatives of the unions were present at the meetings in which the first Action Plan, with an implementation period from 2007 to 2013, was discussed and adopted (see e.g., Ministerio de Trabajo y Promoción del Empleo, 2007b). An advisory representative of the ILO is present in the majority of the sessions.

The second plan, which the ILO drafted and subjected to deliberation by the Commission members, was crucial. It specified the intervening activities in more concrete terms and defined the three focus sectors for 2013 until 2017 (Ministerio de Trabajo y Promoción del Empleo, 2013a). The ILO’s Bridge Project was launched in the country during this period. The issue of including domestic work as a focal point for action was not further discussed by the commission nor have domestic workers’ representatives been present. Immediately after the adoption of the second Action Plan, referring to observations of the CEACR, the ILO urged the Commission “to carry out baseline research or rapid assessment in the area of domestic work, in order to have more elements to implement the strategy to prevent and combat forced labor in this sector.” (Ministerio de Trabajo y Promoción del Empleo, 2013b, own translation). It appears that the Commission has neither addressed the issue nor passed a resolution on it. Additionally, the CEACR reported that a lack of funding hindered the implementation of the second National Action Plan (ILO, 2021a). The third plan reaffirmed the governments’ commitment and the previously agreed measures for another 5 years until 2022 (Ministerio de Trabajo y Promoción del Empleo, 2017). The Action Plans were drawn up in close co-operation with the ILO, they refer to ILO normative standards and reports and are “not only inspired by ILO norms but also their activities carried out in the context of forced labour.” (Thomann, 2011, p. 277).

Another outcome of institution building is the legislation. The ILO Bridge Project’s coordinator was a legal expert and heavily involved in the negotiations of new legislation. As a result of the ILO’s legal advice and technical meetings, a law was passed in 2017 (Ley Código Penal (Art. 168-B)) which made forced labor a criminal offense and is closely aligned with the Convention No. 29. The interviewees indicated that there is still a noticeable absence of references to the Convention and national legislation on this matter in legal proceedings or in cases of severe labor rights violations reported by trade unions to the police, particularly with regard to domestic work. During the COVID-19 pandemic, in 2021, campaigns were conducted with domestic workers urging the Peruvian government to ratify the ILO’s 2014 Protocol to Convention No. 29. According to this legal document, the ratifying member states commit themselves to the identification, release and protection of victims of forced labor in order to achieve their reintegration, providing them with some form of assistance (ILO, 2014). The protocol was ratified in Peru in the same year, 2021.

These three institutions serve as a legal and political structure, defining the frame of reference for the observation and persecution of coercive labor relations. The absence of domestic workers’ organizations in the Commission likely influences the discussion and thus the direction of policy measures.

#### 4.2.3 Information campaigns and educational training programs

Information and training material on forced labor, in particular its definition and indicators, was requested from the ILO by the Commission – which indicates that there was no previously established concept in Peru. This endeavor requires a transfer of the forced labor category and its interpretative patterns to the Peruvian context in order to reach the public. Local adaptation is essential for delivering messages in understandable formats through appropriate channels (see Merry, 2006a, p. 158). For example, the incentive to have the ILO create flyers with comic stories on forced labor, in order to reach a wide range of workers, came from the worker’s union representative in the Commission (Ministerio de Trabajo y Promoción del Empleo, 2012). With the publication of a handbook for the prevention and identification of forced labor for justices of peace (OIT, 2018) in Spanish and in the indigenous Aymara language, the ILO not only adapted the language to certain areas of Peru (especially the Andean and Amazon regions). By addressing and adapting the handbook to the specific group of justices of peace, the specificities of the communal justice systems relevant to the persons concerned were taken into account. In the area of domestic work, the ILO’s Bridge Project started to carry out information campaigns and training program with trade unions in 2017. The ILO chose various channels of communication, among them radio, flyers, videos, handbooks and workshops. In order to generate awareness and mobilize action, it is essential that the communication resonates with local terms, practices and understandings while remaining aligned with the framework of the international category (Levitt and Merry, 2009, p. 451, 452). That communication takes place in a tension between the universal, institutionalized language of the ILO and national, local and sector-specific terms can be observed in the different media outputs and the resonance among domestic workers.

Exemplary short stories of forced domestic work and brief information broadcasts were specifically created for radio stations in different languages – such as Spanish and Aymara – to reach rural-born domestic workers at their home or workplace. Among other things, popular Andean music and rhythms were used as a stylistic device. Considerable effort went into a radio and successful video format with the attention-grabbing title “story of a victim of modern slavery in Peru” published in 2018 (Oficina de la OIT para América Latina y el Caribe, 2018). The experience of the fictional character Julia serves to illustrate a typical pattern of deceptive recruitment and coercive working conditions for young girls and women from rural areas working in urban households. The actor’s hairstyle with two braided plaits indicates Andean culture. For the production of this story, the ILO shared the draft and consulted the trade unionists.

*“To be able to be close to reality. So that the product really reaches the people it is meant to reach. So that it represents reality as much as possible, the reality of what is happening. The language, even the accent of the area.”* (ILO communications consultant).

According to the ILO staff, such processes of consultation and interaction with trade unionists not only allow for feedback from the workers but are intended “to validate the products and messages with counterparts and implementing partners, so that they take ownership of them and disseminate them through their own communication channels.” (ILO communications consultant).

These consultations thus strategically ensure the distribution and visibility of the topic and the ILO programme, though this procedure was not carried out for every publication, or, in some cases, the perspective of the domestic workers’ unionists was not integrated in the final product. The latter was the case for a series of videos produced by the ILO in 2021, in which an external consultant explains the definition and indicators of forced labor in domestic work. Since the domestic workers did not agree with the explainer and style of the format, the video was never shared on their own platforms and was hardly viewed on YouTube (ILO, 2021b). The ILO’s subsequent animated video series in 2023 again used a storytelling approach but this time included a domestic worker trade unionist character. The series has a clear learning style, stating “learn with Rosa what forced labor is and what she can do to change her situation, with the support of the trade unions.” (ILO, 2023). That this format includes a trade unionist and explicitly shows their role in the fight against forced labor as well as mechanisms that can be followed for practical support, legal action and prevention conveys a message of agency and partnership regarding the unions. All published videos on YouTube include logos of domestic workers’ trade unions to indicate their involvement in the production (regardless of how closely they were integrated in the process).

Other materials have a more representative character toward international cooperation. For example, the guidebook on identification, prevention and persecution of forced labor in the domestic work sector is directed at trade unions and translates the ILO’s indicators of forced labor to the Peruvian context of domestic work (OIT, 2018). The publication employs a language format according to the UN’s Sustainable Development Goals and ILO’s legal language which maintains international legitimacy and is presentable to donors and the government (see OIT, 2018) but is of less practical benefit for the domestic workers’ unions. Workshops on the guidebook’s content were held in 2018 with domestic workers’ trade unions in urban centers of the country, in the capital Lima and Chiclayo, capital of the Lambayeque region in the north-west. The ILO’s eleven indicators of forced labor^23^ were adapted to the sector of domestic work. For example, regarding the indicator ‘restriction of movement’, the explanation explicitly refers to a house as the workplace and all indicators refer to a female worker (OIT, 2018, p. 27). Because of their social and institutional position as a highly educated, middle−/upper-class international political elite, ILO specialists, in encounters with the organized domestic workers, “do not have intuitive access to the local cultural toolkit, which might make new ideas and practices more accessible and attractive.” (Rajaram and Zararia, 2009, p. 481). Even though the ILO specialists might also be Peruvian born, the social distance to their counterparts who used to be child workers and were often brought from rural areas to the coastal cities presents a challenge in the bridging of “transnational ideas and local concerns” (Merry, 2006a, p.134).

### 4.3 Appropriation of the category and trade unions’ mobilization

The trade unionists are addressed by the ILO as multipliers and mediators, rather than as affected domestic workers. They themselves should be the ones who act as translators and translate the learned discourses, framings and practices of international labor law and the ILO to local situations in their own institutional spaces (e.g., in counseling, legal proceedings or campaigns), thus translating or reframing the local manifestations of violent and coercive labor into the ILO’s category of forced labor.

The prominent studies on processes of vernacularization, the adoption of human rights norms in local contexts, describe various roles that actors take on in the process (see Merry, 2006a; Merry and Levitt, 2017; Rajaram and Zararia, 2009). Here, mediating actors between the state or international organizations and those affected are primarily described as middle-class, higher educated persons who move in transnational spaces (such as UN conferences) as well as in local spaces and translate between them. Unlike these examples, the trade unionists in this present case study are not middle-class, institutionally higher educated persons. They are domestic workers themselves and also have transnational organizational experience (e.g., in the International Domestic Workers Federation IDWF) as well as experience in transnational political spaces as delegates (e.g., in the ILC). They are actors who move between the spaces and therefore “move between discourses of the localities they work with.” (Merry, 2006a, p. 39). This particularity harbors a special potential for influencing non-organized domestic workers. To access the transnational discourses and concepts, Peruvian domestic workers depend on their organized colleagues, who themselves depend on international organizations like the ILO or the IDWF.

In contrast to the ILO specialists, the trade unionists have a double perspective in the process of vernacularization. On the one hand, they are “enactors of vernacularization” (Levitt and Merry, 2009, p. 449), on the other hand, they themselves are the addressed recipients and to some extent survivors of coercive and violent labor relations. The fact that they themselves are locals and part of the affected group of domestic workers gives them a credibility and power of communication that the ILO specialists do not have. As domestic workers and trade unionists, they describe their own experiences, observations and interpretations, whereby the nationally institutionalized ILO category of forced labor serves as a frame of reference only to a limited extent. Domestic workers, in the role of delegates of their trade unions, were not part of the policy plan and legal reform negotiations but are addressed as actors in prevention measures by the ILO Bridge project and by the government’s National Action Plans.

#### 4.3.1 New framing for well-known issues

The unionists and activists are confronted with violent and coercive labor relations in the sector on a daily basis, but they have not referred to these situations as forced labor.

*“Here there is no talk of forced labor or trafficking. […] For the compañeras, they do not see it as forced labor or trafficking. Even though many of the life stories of the compañeras who have come here, their mothers have given them to a woman, and they are here, and they do not know their parents. They have worked since they were little. They have been taken to a house, they have been mistreated, they have been beaten, they have been raped. And at the end - I mean, they made them work and they did not pay them. There are many such cases.”* (Sirena O., Trade Unionist and Activist).

*“But in Peru, even though there is a law, it is not common to talk about forced labor. It’s not when you get fired from a job and you want to collect your benefits. It’s not when you suffer rape and harassment. Many times, you do not report it. For example, we in the Federation […] have not made any reports about forced labor.”* (Delia M., Trade Unionist).

The problem complex is not new to them, framing it as forced labor is. In ILO publications that established forced labor as a global phenomenon of social inequality in the early 2000s (ILO, 2001; ILO, 2005a), it is referred to as the “underside of globalization” (ILO, 2005a, p. 63) in the private economy. This framing as a negative effect of globalization differs from the local framing of violence in domestic labor in Peru made by the organized domestic workers. They frame the degrading relations, devalued role, labor rights violations, and harmful treatment of domestic workers, particularly those from Black and indigenous communities, as a continuation of social injustice in colonial-patriarchal labor and gender regimes.

*“Our reflection is that Peru became independent from the Spanish Empire, but we, the domestic workers and the people, have not been emancipated. […] We Domestic Workers still find ourselves in a situation of semi-slavery in a modern society.”* (FENTRAHOGARP, 2021c, p. 2).

Historically, the justice theory and strategies of the domestic workers’ organizations are embedded in liberation theology (cf. Mujica and Meza, 2009),^24^ which, in the name of social justice, advocates the emancipation of the poor and the restructuring of (economic) power structures. In this perspective, liberation is conceptualized not only in terms of the afterlife, but also as the political and existential liberation of the lower classes, by the lower classes (Gutiérrez, 1973). It promotes collective organizing instead of individualized charity. The Young Christian Workers of Peru (JOC)^25^ went to night schools and directly approached domestic workers who came from rural regions and wanted to reach basic or higher educational levels. A leader of the domestic workers’ movement remembers:

*“And there the JOC did the work in the evening schools. […] And they told us ‘Commit yourself to work for the others, for the other domestic workers, support the others, train yourself in all aspects’. They trained you in politics, in women’s issues, in rights, in the law. Everything. So, that’s how we formed our own union in our group, whilst we were also members of the JOC.” (Lorena L., Trade Unionist and Activist).*

The trade unionists already use rights language with their clients to whom they provide legal advice concerning the Peruvian labor law, which they themselves negotiated. Establishing a rights consciousness among paid domestic workers is a challenge for the activists since it clashes with normative ideas of kinship reciprocities, family duties and productive work. Often, the workers are related to the employers or otherwise connected in a community. Enduring mistreatment is therefore mostly interpreted as part of “kinship obligations” (see Pérez and Freier, 2020) and is not classified by affected persons as violence or a violation of rights. Addressing domestic workers as ‘part of the family’ disguises the paternalistic hierarchical power relationship in the household (Schalkowski, 2024; Anderson, 2000). To a certain extent, the trade unionists break with the norm of the primacy of the “integrity of the family” (Rajaram and Zararia, 2009, p. 473) and try to establish the perspective that the private sphere of the household is a workplace which includes institutionalized rights and duties. Moreover, they challenge the concept of the household sphere as a private domain where the state does not intervene.

Classifying severe conditions in domestic work as forced labor, or generally as violent, is not an established pattern of interpretation in Peru. Violence against domestic workers (especially racialized women) is normalized and met with indifference by authorities in Peru (Blofield and Jokela, 2018; Maich, 2014). This is one of the reasons for domestic workers’ organizations to resort to “globally available discourses” (Rajaram and Zararia, 2009, p. 481), which the ILO offers with its internationally institutionalized legal categories and indicators. Independently of forced labor, this applies to the ILO Convention No. 189 on standard setting for market-based domestic work, which was largely advanced by Latin American trade unions (Blofield and Jokela, 2018, p. 538). Action was taken at the transnational level in order to invoke these instruments and resources at the national level and thus exert pressure on governments. By framing and mobilizing their labor rights struggle consistent with ILO norms and concepts, the workers’ activists gain political legitimacy to which state actors are more responsive, and they can reach a broader (including international) audience and facilitate alliances.

#### 4.3.2 Self-identification

The ILO’s category opens up a new possibility of meaning-making that enables workers to classify their experience not only as labor rights violations but as legally punishable coercion and violence. This can influence the self-perception of a person. After participating at ILO training on the identification of forced labor, trade union members self-identify using the ILO indicators:

*“Now I understand that I was in a forced labor. Why was that? Because I wasn’t paid. I was locked up. They gave me the used clothes of the children of the Señores.*^26^*”* (Delia M., Trade Unionist).

*“It’s only when they do a workshop that we realize, is not it? And then that’s when one says ‘ah, this is what happened to me, this is it’. That’s where you know. But the only ones who have found out, are those of us who have been there.”* (Sirena O., Trade Unionist and Activist).

In order to identify one’s experience as forced labor and oneself as a person subjected to forced labor, one first has to identify as a worker – which is not necessarily the case in the field of domestic work, due to its informal organization and its naturalization as a (racialized) women’s responsibility within the private sphere of a family. It is also important to highlight that the interactions concerning the mobilization against forced domestic work take place between the ILO and trade unions which consist of market-based workers in paid domestic work. Arrangements in the household that include reproductive tasks – which are not interpreted as an employer-employee relationship, whether informal or formal – are neither represented nor considered.

#### 4.3.3 The category as a campaign tool

The appropriation of the ILO’s forced labor framework by domestic workers unionists was especially prominent in a campaign in 2021. The National Federation FENTRAHOGARP carried out an online and street campaign called #NoMásTrabajoForzoso (No More Forced Labor) demanding compliance with Legislative Resolution No. 31160, which ratified the ILO Protocol concerning forced labor (April 2021). This campaign was specifically related to the implementation of the instrument and was directed not at colleagues concerned, but at the government, while using terms consistent with the ILO’s framework.

They produced their own posts with pictures and videos for social media, with resources from the ILO, but under their own direction. In different video formats, organized domestic workers talk personally about their own experiences and address the mobilization against forced labor and the mistreatment of domestic workers in general:

“*No more forced labor: ‘They withheld my ID card to force me to continue working,’ is the testimony of Alfonsina Manchay, a domestic worker who was a victim of one of the forms of forced labor in our country*.” (FENTRAHOGARP, 2022, 30th of March 30, own translation).

*“‘I was handed over to so-called godparents for a plate of food, I was shouted at and beaten,’ says Inés Meza, a courageous leader of the Domestic Workers, who welcomes the progress made to put an end to forced labor.”* (FENTRAHOGARP, 2021a, 26th of April, own translation).

*“#Nomoreforcedlabor: The historical leader, Adelinda Diaz, with her testimony and life story, seeks to raise awareness about the situation that Domestic Workers are going through in our country.”* (FENTRAHOGARP, 2021b, 17th of April, own translation).

The experiences are classified as forced labor or even slavery (without making clear how the two concepts are distinguished). The unionists emphasize common experiences and establish connections between the socio-cultural arrangement of the labor relationship and the framework of forced labor. With the occasional appearance of the term slavery, they establish, on the one hand, a historical connection to the experience of domestic servitude (see Schalkowski, 2024), and on the other, the term has a scandalizing effect, e.g., in this statement: “Forced labor is part of the slavery of women workers.” (FENTRAHOGARP, 2021b, April 17). The speakers are positioned in front of screens with the union logo and hold hand-painted posters in their hands with statements against forced labor. This format simultaneously emphasizes the collective experience as well as the individual story of the worker, who tells it in her words and creates a poster with her own writing. One video is accompanied by the sound of a women singing about her story of arriving in Lima as a domestic worker (FENTRAHOGARP, 2021b, 17th of April). The informational posts, with large lettering and sometimes with pictures of protest actions, are designed in the organizations’ own style, but the wording is very similar to that of the ILO’s explanation of the definition and indicators of the forced labor category.

In interviews with the trade unionists and the ILO, it became clear that the trade unions know how to use the ILO as a resource and instrument in their political struggle. They follow the logic of the ILO activities and norms to some extent. An insightful comment is made by a trade unionist who identifies the ILO as the initiator of campaigns against forced labor in domestic work. She also recognizes a lack of sustainability of such activities and ILO information campaigns.

*“Look, FENTRAHOGARP took advantage making this campaign because they were giving support as well. There at the ILO. Giving support so that they could carry out this campaign. Because the union did not have any money. And so, they were giving support for this campaign.”* (Sirena O., Trade Unionist and Activist).

*“When, for example, the ILO brings something up, such as forced labor. The ILO brings it up. And they start campaigning with the unions, with the organizations. And once in a while it’s on TV. […] But after that, nothing. Nothing. Nothing. So, it’s like those campaigns are at that moment, but afterwards, I mean, it’s not a campaign that has continuity.”* (Sirena O., Trade Unionist and Activist).

Regarding the ILO’s training activities and encounters with the domestic workers’ organizations, on the one hand, the activists are requesting more continuity to be able to incorporate the concepts in their daily practice. On the other hand, the institutional and economical power asymmetries between the ILO specialists and the domestic workers, which determine the organization of these interactions, are criticized:

*“And sometimes in the projects they do here for the ILO, […] they earn their big salaries and the domestic worker, practically, we have to go to the training, to the events that they are doing. They also support us, and sometimes they say ‘you are part of the training, the tasks you have to do, you have to keep working, because there is no budget for salaries, but there is a budget for activities.’ […] But so far, we have done it with pleasure, with money, without money.”* (Lorena. L, Trade Unionist and Activist).

So far, the social and legal services provided by the domestic workers’ organizations have not been adapted or expanded concerning forced labor, for example with special care for victims of forced labor, or a specialized legal advisor. Also, until now, there have been no attempts to obtain government funding or to interact with the established institutions (e.g., the Commission) in the field of forced labor. It is the ILO which is more likely to be approached by the organizations concerning funding or technical expertise.

It is hard to say what translation process has taken place between the participants of ILO workshops, especially those in leading positions in the unions, and their organization or their counseling cases. Since there have been hardly any reports by the organizations to the police or labor inspection on forced labor in the sector, it suggests that the category has not been included in the counseling processes as a horizon of possible (legal) framing so far. At most, the topic is raised rhetorically as an issue on the political stage, usually only in connection with the ILO. Trade unionists as well as labor lawyers, advisors and social scientists point to a lack of knowledge about forced labor and a lack of practical experience with the new legislation which hinders its application.

## 5 Discussion

The mobilization against forced domestic work is part of the vernacularization of the ILO’s forced labor category into Peruvian contexts. Peru is a field of experimentation for the ILO’s SAP-FL program and serves as an empirical example of mobilizing a labor norm at the “interfac[e] between national-local and international scenes” (Kott, 2011, p. 450). The case of forced domestic work as a political field of action in Peru is also instructive for sociological debates on categorization and observation schemes that determine knowledge constructions and frame policies – especially since the active role of actors in their historical-cultural normative contexts is elaborated. The use of the theoretical lens of vernacularization, a contextualizing examination of the labor policy actors, their positions and knowledge bases, as well as an analysis of decisive moments, spaces, technologies and developments of communicative actions, enables a sociological understanding of the emergence and manifestation of the mobilization against forced domestic work. It is in the interaction between the ILO and labor policy actors that the translation and localization of the abstract category is realized.

On a political, legal and trade union level, the mobilization in Peru takes place, on the one hand, within the reconfiguration of the domestic work sector since the early 2000s. The institutionalized manifestations of the national and international movement of domestic workers are the ILO Convention No. 189 on Domestic Work (ILO, 2011a) and the renegotiated Peruvian labor legislation regarding the sector (2021). On the other hand, the mobilization against forced labor is part of the internationalization of the ILO’s renewed policy script and its underlying category of forced labor. The topic gained momentum during the COVID-19 pandemic, when cases of severe labor rights violations were publicly discussed and scandalized. The ILO’s category is a visible reference point of the trade union campaign against forced labor and of the Ministry of Labor’s National Action Plans. Legal and political communication about coercion- and violence-based labor in domestic work is carried out in the terminology of the ILO. Although the category of forced labor is concretized through vernacularization, the implementation and adaptation of the category follows a rather pragmatic approach, which simplifies complex local manifestations and articulations of the phenomenon into universalistic indicators. The ILO’s framework, which is embedded in the normative concept of free wage labor (distinguished from slavery),adds a new dimension to the social justice framework established by trade unions, which challenges the prevailing local norms concerning private households, families and labor relationships. The trade unions challenge these arrangements of labor which they understand as a continuity of colonial-patriarchal labor regimes. The ILO’s observation and categorization patterns of (forced) labor are confirmed in the adaptation of the category and direct national policy and trade union mobilization against forced domestic work.

The vernacularization approach has shown how the category and its patterns of interpretation of labor were translated into the Peruvian context of paid domestic work and introduced to local labor policy actors – in particular domestic workers’ trade unions. Through mobilization of the trade unionists, the ILO influences national labor policies and reform processes (see Koliev, 2022, Plata-Stenger, 2020, p. 215). For these activists, framing coercive and violent labor relations in their sector as forced labor means reinterpreting the experiences of the workers in politically and legally more powerful, internationally legitimatized, terms in order to claim their rights and ultimately achieve a transformation of domestic work arrangements. Aligning their struggles with the international development cooperation’s fight against forced labor allows them to mobilize support from international organizations and advocates. The trade unionists use the category as a political campaigning tool (to a small extent) and know how to use the ILO as a resource and instrument in their political struggle. For them, the category of forced labor can serve as a political and legal communication tool. Certain relations in domestic work can be categorized as forced labor based on the ILO indicators and the labor law (which is based on the ILO definition), and can be communicated in this way, e.g., in court or in connection with political campaigns and demands. The term is therefore more than just a scandalizing epithet, as it can be used to refer specifically to established legal standards. At the same time, the category has an effect on the actors themselves – they are given a different framework to reflect on their own situation or experiences and now see themselves in some cases as victims of forced labor.

For the ILO, I argue, in addition to the internationalization of its labor standards within a development policy context, technical assistance within the framework of public policy programs in Peru has a heuristic value. By collecting information on local manifestations of forced labor, knowledge about the matter is produced and the ILO’s lens is sharpened as a result. For the implementation of their technicized (partly abstract) knowledge, the ILO relies on local actors and their expertise and experience. In the case of spatially as well as socially isolated domestic workers (Schalkowski, 2024) in private households, the trade unions are key to access and disseminate information. The ILO’s global program to end forced labor sets the organizational structure, the normative and knowledge framework, “while the local context provides its distinctive content” (Merry, 2006b, p. 44). The vernacularization process is structured by the power hierarchy between the ILO specialists and the trade unionists. Their actions are shaped by various factors, including the sources of their financial support, their ethnic and gender identities, as well as their socio-economic position, and institutional structures (Merry, 2006b, p. 40).

Since vernacularizers operate within certain “discursive fields” (Merry, 2006b, p. 40) that inherently shape and restrict the ideas and practices they can engage with, the gender-specific limitations of the ILO’s category of (forced) labor have a restricting effect and are reproduced within the process. Consequently, “comparison criteria based on non-recognition and exclusion are continuously confirmed” (Wobbe, 2020, p. 148; own translation), even though the interpretive framework can be broadened by local actors.

Which segments of the labor market are even considered for investigation as well as regulation and political action within the specific constructed framework of forced labor has consequences for the configuration of labor policies and labor law – and consequently for the working individuals:

*“Especially for workers in coerced labor situations, it may not be of any interest what kind of definitions are currently favored within specific institutions, but it may be of fundamental relevance to their lives if forms of coercion and exploitation are perceived by other social actors as for example ‘normal,’ ‘criminal,’ or ‘necessary’.”* (Carstensen, 2016, p. 127).

Circumstances like the ever-changing composition of the Peruvian government in recent years, the socio-economic situation of domestic workers (unions), the ILO’s embeddedness in development strategies or the global COVID-19 pandemic have an influence on the social process of translation and adaptation of the international labor norm. Historically contextualizing not only the contingent construction of labor categories in transnational spaces but also contextualizing their translation and adaption, is sociologically productive in order to reflect on the construction of problems and their respective fields of political action. This perspective is reflected in the methodological approach, which differs from historical studies on international organizations and norms as well as from global governance studies which focus on changes in national and international legislation and policy outcomes (cf. Thomann, 2011; Phillips and Mieres, 2015). It included not only historically reconstructing the establishment of the forced labor category on the international and Peruvian national level, but also qualitatively approached involved actors on a micro-level, thereby gaining access to individual and collectively shared interpretations, knowledge bases and communicative dynamics.

## 6 Outlook

As a *double deviation* from the norm, forced domestic work suggests not only complex dependency and power relations within labor and family structures, but also indicates particular patterns of classification of labor and unfreedom.

Behind the debates on modern slavery and forced labor of recent decades (Patterson and Zhuo, 2018) are certain categories and patterns of interpretation of social arrangements of work, their dependencies and social hierarchies, which reveal the historical-geographical context-dependency of perspectives on the subject. In the case of forced domestic work, the categories of forced labor and non-standard employment intersect. As historians have shown, the term ‘standard’ is misleading, since “the real norm or standard in global capitalism is insecurity, informality or precariousness” (Breman and van der Linden, 2014, p. 920). Labor history is often synonymous with the history of wage labor (Haschemi Yekani, 2019, p. 29). In contrast to this Eurocentric normative view of labor, a group of historians examining labor coercion challenges the assumption that ‘free’ wage labor is inherently self-evident. They assume a compatibility between capitalist market structures and coercion-based labor relations. Christian De Vito, Juliane Schiel and Matthias van Rossum propose the following in an article in the Journal of Social History:

*“Neither the free wage laborer nor the male breadwinner model or the capitalist mode of production can form a blueprint for our endeavor. Instead, we address the persistence and transformation of coercion and bondage across world empires, gender regimes, and historical eras to overcome the classic divides of labor history discourse (free/unfree, productive/unproductive, capitalist/precapitalist) by linking the stories of work and production with those of violence, expropriation, marginalization, and criminalization.”* (De Vito et al., 2020, p. 645).

The Western normative framework of a free market with private property, monetarization and free wage labor became the trademark of European civilization and progress (Cooper, 2019). In line with scholars that question this idea as an applicable standard, Magaly Rodríguez García (2016) states that “a radical distinction between free and coerced labor obscures the fact that constraints are inherent in all types of labor relationships.” (Rodríguez García, 2016, p. 13; see also Steinfeld and Engerman, 1997). Geographer and feminist political economist Kendra Strauss is among the scholars who call for an understanding of unfreedom as a continuum and examines how modes of production stand in relation to forms of unfreedom (Strauss, 2012). Even when work is carried out in the harshest conditions, there is no debate in sociology about the conditions under which work can be called ‘free’, as this is assumed to be the norm in our modern society and is perceived as synonymous with wage employment. Interpretations and categorizations of activities as ‘forced labor’ have so far been little discussed. This is especially true with regard to feminized and racialized activities that are associated with being non-productive and therefore not considered as work in all contexts, − such as domestic work. Strauss points to the richness of this field for conceptual discussion: “domestic labour and the household are complex sites of interlocking relations of unfreedom.” (Strauss, 2012, p. 193).

In general, there is a lack of sociological-conceptual approaches to coercive and violent (labor) relations that go beyond established legal categories and the simplistic binary of free versus unfree labor, focusing instead on socio-cultural power dimensions and not only on economic aspects of exploitation (see Patterson and Zhuo, 2018; Schalkowski, 2024).

Future research as well as political instruments must address both market-based and non-market-based forms of domestic labor to ensure a comprehensive analysis in knowledge production to provide orientation for policy action on governmental and labor movement levels. In the field of political economy, scholars apply an instructive lens of social reproduction theory to research entanglements in coercive labor, going “beyond economistic emphasis on the productive sphere to explore the ways that households and reproductive activities and relations shape labor relations and conditions.” (LeBaron and Gore, 2019, p. 575).

Regarding the political implications of the studies’ results, several points can be highlighted. The ILO’s framework concerning forced labor and its indicators are more likely to not only be recognized as a classification concept but also to be incorporated in daily practices of local labor activism, if it “build[s] on local movements of resistance and contestation.” (Merry, 2006a, p. 100). Furthermore, the implementation of the forced labor category is only relevant as a potentially transformative resource for domestic workers if institutional structures reinforce the norm and are sustainably funded. Negligence and other negative experiences of encounters with authorities or legal judgments can hinder the adoption of these instruments, as they may not be perceived as effective by affected persons and activists (Merry, 2006a, p. 222). This can already be observed in the context of the legal regulation of domestic work in Peru which is largely met with indifference by employers as well as state authorities (Blofield and Jokela, 2018; Maich, 2014). Efforts to reform legislation and change social practices of coercive labor relations lack an analysis of their embeddedness in the historically built socio-economic gender and capitalist structures that maintain them. In the words of legal scholar Adelle Blackett: “Regulatory frameworks will be unenforceable, and will fail to formalize domestic work, if they are not attentive to the existing norms that order the relationship in highly inequitable ways.” (Blackett, 2020, p. 111).

In this regard, the discourse on structural violence against (racialized) women and girls has also not sufficiently addressed these degrading practices. The importance of trade unions and domestic workers’ organizations in the fight against gender violence has been largely undervalued.

## Data Availability

The datasets presented in this article are not readily available because the raw dataset consists of interview material which includes information that compromises anonymity of participants and can therefore not be shared publicly. Requests to access the datasets should be directed to n.schalkowski@fu-berlin.de.

## Footnotes

^1^The term ‘forced labor’ refers to the International Labour Organizations’ legal category defined in the Convention No. 29 (ILO, 1930) and is not used as an analytical category in this article.

^2^Although the Peruvian Labour Law No. 27986 recognized and regulated domestic work for the first time in 2003, the regulations remained far below the national standard for other sectors.

^3^This paper is part of the collective project ‘Forced Labour as a Shifting Global Category: Classification, Comparison and Meanings of Work in the International Labour Organization (ILO), 1919–2017’ (FU Berlin, PIs: Marianne Braig and Theresa Wobbe), funded by the Deutsche Forschungsgemeinschaft (DFG Grant No. 413607635).

^4^The ILO’s tripartite governance model includes governments, as well as organized workers and employers in the decision-making processes.

^5^International labor regulation segmented the standard employment relationships’ “central pillars” (Vosko, 2009, p. 62).

^6^The ILO regarded ‘colonial labor’ as a distinct category, specifically termed ‘native labor’ which was subject to its own set of regulations and standards (see Maul, 2007, p. 481).

^7^Asociación de Mujeres Negras Trabajadoras del Perú.

^8^Federación Nacional de Trabajadores y Trabajadoras del Hogar Remunerados del Perú.

^9^Federación Nacional Trabajadoras y Trabajadores del Hogar Perú.

^10^Instituto de Promoción y Formación de Trabajadoras del Hogar.

^11^Sindicato de Trabajadoras y Trabajadores del Hogar de la Región Lima.

^12^Sindicato Nacional de Trabajadoras del Hogar del Perú.

^13^ILO Bureau for Workers’ Activities.

^14^Employment agency and labor rights training for domestic workers in Lima.

^15^Feminist organization in Lima that delivers annual reports to the UN on the situation of women.

^16^Asociación Nacional de Trabajadoras del Hogar.

^17^In 1963, the headquarter of the ILO Regional Office for Latin America started operating in Lima. It therefore has a special status and substantial resources for technical cooperation in the country.

^18^Marieke Louis and Lucile Maertens argue that international organizations aim to be seen as neutral, technical, and objective entities rather than political organizations, in order to maintain legitimacy and ensure cooperation (Louis and Maertens, 2021).

^19^The ILO’s Bridge Project followed a binational cooperation project “Consolidating and Spreading Efforts to Combat Forced Labour in Brazil and Peru” (2012–2016, funded by the USDOL).

^20^This qualitative study was embedded in the ILO’s global initiative to collect data on the issue within the SAP-FL (see Schalkowski and Renard, 2024).

^21^Concerning the entanglement of productive and reproductive work, including unfree forms of work in extractivist sectors, see Cabrapan and Duarte (2019): Género, sexualidades y mercados sexuales en sitios extractivos de América Latina; Dunaway (2014): Gendered Commodity Chains. Seeing Womens’s Work and Households in Global Production; LeBaron and Gore (2019): Gender and Forced Labour: Understanding the Links in Global Cocoa Supply Chains.

^22^In 2022, the ILO published a study on the pandemic’s impact on risk factors for forced domestic labor, based on questionnaires and other data (OIT, 2022). A planned household survey on forced domestic labor in Cusco was postponed indefinitely due to pandemic-related restrictions and the change of government.

^23^The indicators include: Abuse of vulnerability, Deception, Restriction of movement, Isolation, Physical and sexual violence, Intimidation and threats, Retention of identity documents, Withholding of wages, Debt bondage, Abusive working and living conditions, Excessive overtime (ILO, 2012).

^24^Liberation theology became prominent in 1970s through the Peruvian priest Gustavo Gutiérrez and spread across the continent as a new theological movement (see Zegarra, 2023).

^25^Juventud Obrera Cristiana del Perú.

^26^Employers of the household.
